# Towards a New Integrated Model for Taking Into Account the Experiential Knowledge of People With Chronic Diseases, Integrating Mediation, Therapeutic Education and Partnership: The Expanded Chronic Care Patient–Professional Partnership Model

**DOI:** 10.1111/hex.70054

**Published:** 2024-10-07

**Authors:** Marie‐Pascale Pomey, Béatrice Schaad, Aline Lasserre‐Moutet, Philip Böhme, Mathieu Jackson

**Affiliations:** ^1^ Research Centre of the University of Montreal Hospital Centre Montréal Québec Canada; ^2^ Centre d'excellence sur le partenariat avec les patients et le public Montréal Québec Canada; ^3^ Department of Health Policy, Management and Evaluation, School of Public Health University of Montréal Québec Canada; ^4^ Department of Family Medicine and Emergency Medicine University of Montréal Montréal Québec Canada; ^5^ Institut des Humanités en Médecine du Centre Hospitalier Universitaire Vaudois (CHUV) Lausanne Switzerland; ^6^ Centre sur le vécu des patient.es et des professionnel.les Direction générale du Centre Hospitalier Universitaire Vaudois (CHUV)/Faculté de Biologie et de Médecine de l'Université de Lausanne (UNIL) Lausanne Switzerland; ^7^ Centre d'éducation thérapeutique du patient Hôpitaux Universitaires de Genève Genève Switzerland; ^8^ Department of Endocrinology, Diabetology and Nutrition CHRU Nancy Nancy France; ^9^ University of Lorraine, Inserm, NGERE Nancy France

**Keywords:** Chronic Care Model, coproduction, integrated care, mediation, patient partnership, therapeutic patient education

## Abstract

**Introduction:**

The Chronic Care Model (CCM), the Expanded Chronic Care Model (ECCM) and the eHealth Enhanced Chronic Care Model (eCCM) focus on how healthcare teams and eHealth support can offer effective care and relevant solutions for patients facing chronic care conditions. However, they do not consider how patients can help these teams in their work, nor do they promote ways in which patients can help themselves. However, in the last decade, three different models have emerged that can complete our capacity to design and deliver integrated care for people with chronic diseases. In this article, we propose a revised version of the model that integrates the patient perspective and patients' experience‐based knowledge. It integrates three different ways of engaging patients that complement the other patient engagement point of view: the experience of care and mediation in healthcare, therapeutic patient education and patient learning pathways, as well as patient–professional partnership.

**Methodology:**

For each of the three models, we conducted a review of the literature using CINAHL, Medline, OVID, EMBASE PsychINFO, Science Direct and government reports on patient engagement and partnership with their healthcare providers, to integrate the different components of these models into the ECCM and eCCM. The goal is to create a model that better takes into account the experiential knowledge of patients and citizens throughout its different dimensions.

**Results:**

We identified 129 papers based on their framework, design, sample, measures and fit with patient engagement and chronic illness and added our own research when relevant. Integrating the three models provides an opportunity to amplify the role played by the patient perspective in the management of chronic disease. The Expanded Chronic Care Patient–Professional Partnership Model (E2C3PM) is intended to rebalance power relations between healthcare professionals and patients (and their caregivers). This new model is based on recognizing patients' experiential knowledge and their roles as caregivers and as full members of the care team. Integrating patient empowerment into the E2C3PM underscores the importance of coproduction care with patients at the clinical, organizational and system levels within a supportive environment.

**Conclusion:**

Applying this new model should make it possible to better take into account the complexity of chronic diseases, improving the integration not only of care, services and eHealth support but also the various determinants of health and reaching a mutually beneficial settlement among all actors involved.

**Patient or Public Contribution:**

A patient‐researcher contributed to the development of the protocol, the data collection and the preparation and writing of this manuscript.

## 1 Introduction

Patient engagement (PE) is internationally recognized as a key ingredient for improving the healthcare system, such that it is tending to become the ‘new normal’ [1]. As stated by the WHO: ‘Engaging patients and families is equally important in all countries across the world, although the relative priority placed on this concept and the manner in which it is done still differs widely at present’ [2]. Carman et al. note that PE has been called a critical part of a continuously learning health system, a necessary condition for the redesign of the healthcare system, the ‘holy grail’ of healthcare and the next blockbuster drug of the century [3]. So the definition of PE may vary, depending, in particular, on the level of patient involvement in healthcare [4]. However, PE may be consensually defined as ‘the actions people take for their health or to benefit from healthcare’ [5]. As stated by Coulter, ‘patients have an important role to play in their own healthcare’ [6]. Due to their position in the system, they perceive malfunctions as discontinuity of care, workflow failures or broken promises regarding treatments or interventions [7]. Defined as ‘blind spots’ by Reader, Gillespie, and Roberts [8] and Schaad et al. [7], these dysfunctions—not easy to capture due to other monitoring tools such as satisfaction surveys—result in a significant deterioration of the patient's experience of care. PE is widely recognized as being a key component when developing healthcare tailored to patient needs and optimizing quality and safety [9, 10]. This is all the more relevant for people with chronic illnesses, as they develop experiential knowledge of living with illness and using healthcare systems, which is too often underutilized by healthcare systems.

Moreover, in chronic disease management, neither the reference Chronic Care Model (CCM) [11] nor its improved Expanded Chronic Care Model (ECCM) [12] considers patients as actors who can contribute knowledge, skills and know‐how to be mobilized to improve their situations [13]. This article proposes a rereading of the CCM that better takes into account patients' contributions to chronic disease management and contributes to a better integrated model of care [14].

This article examines the challenges faced by the CCM, ECCM and eHealth eCCM and the evidence for incorporating patient experience and knowledge into the CCM. It presents a new model that includes aspects of PE on the same level as professional engagement.

## 2 Challenges of the CCM, the ECCM and the eHealth eCCM

**Figure 1:**
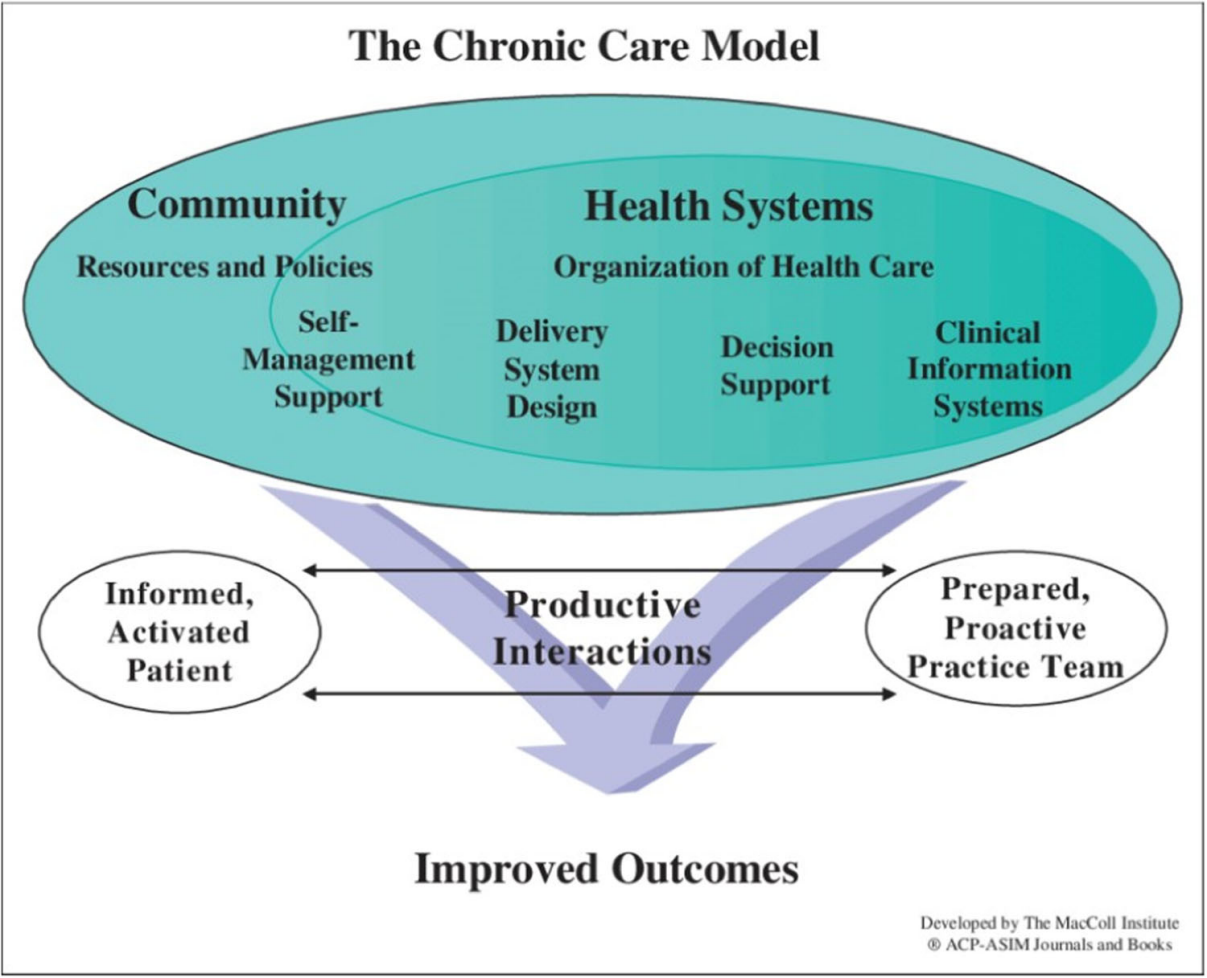
The Chronic Care Model [15].

The CCM was proposed by Wagner in 1998, who showed how, by taking into account various productive interactions between informed, activated patients and a prepared, proactive practice team of clinicians and healthcare professionals, it is possible to improve functional and clinical outcomes in disease management, especially for chronic disease (cf. Figure 1) [16]. This model has been criticized on several occasions, particularly for its inability to adequately encompass or describe the strategies needed to effectively promote health and prevent disease at the population and individual levels [15, 17].

**Figure 2:**
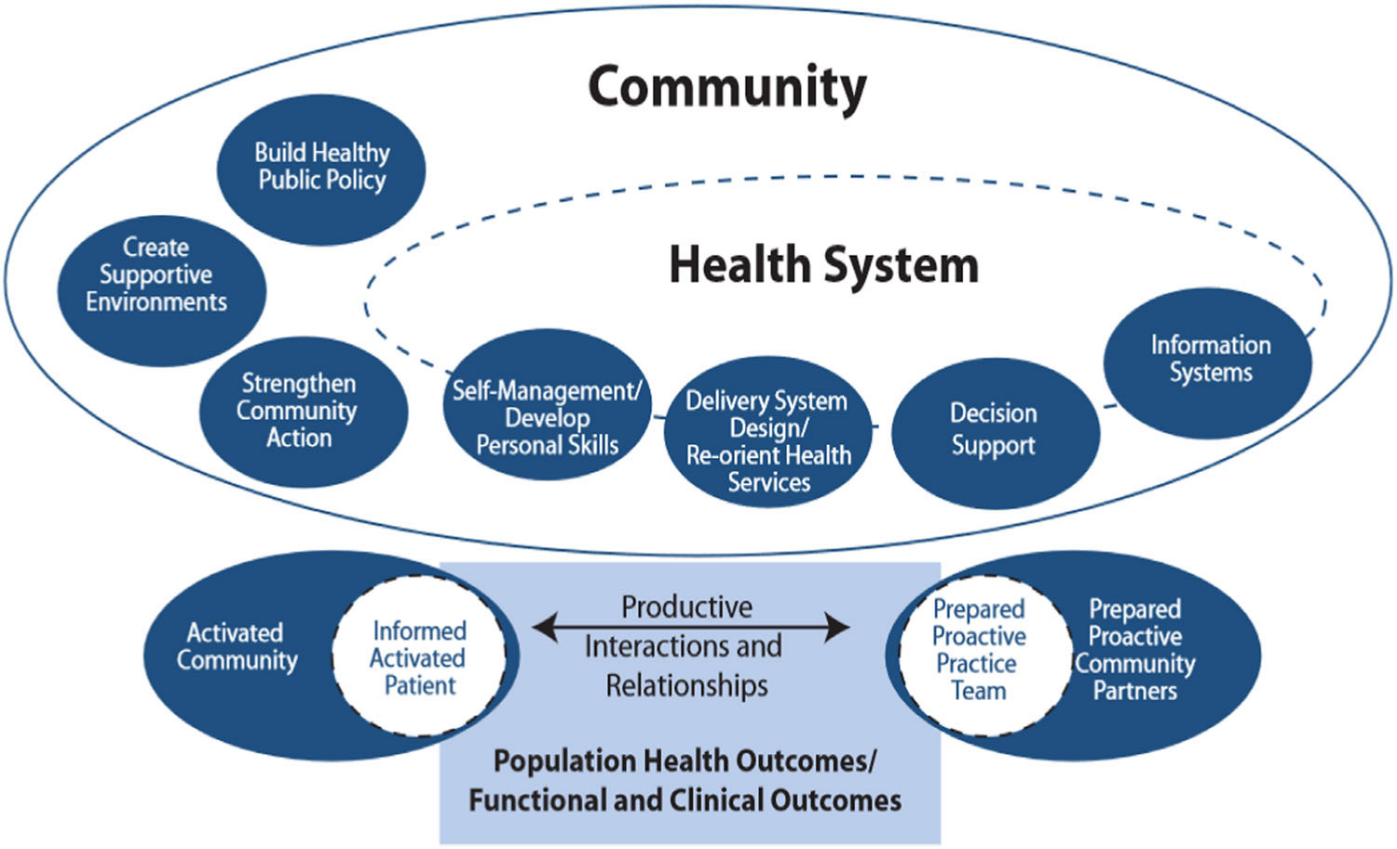
The Expanded Chronic Care Model: integrating population health promotion [12].

To emphasize the healthcare system to address the social, environmental and cultural factors that affect health, Barr et al. proposed a revised model integrating the Ottawa Charter, which recognizes the importance of working on ‘the process of enabling people to increase control over, and to improve, their health’ [12]. This new model, the ECCM, integrates social determinants and population health promotion into the prevention and management of chronic disease (cf. Figure 2).

**Figure 3:**
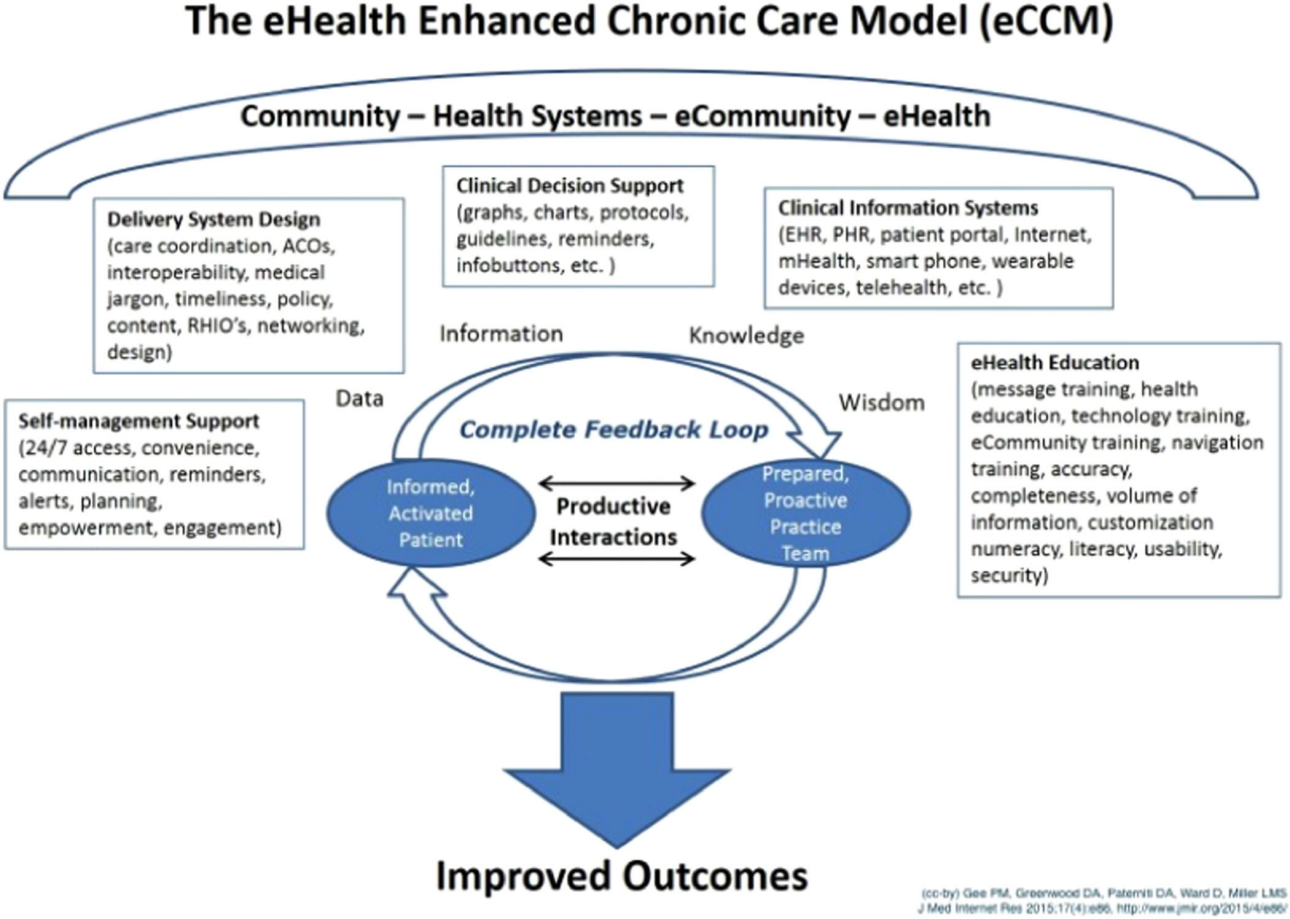
The eHealth Enhanced Chronic Care Model (eCCM) [20].

Even if the CCM and the ECCM take into consideration the central role played by patients in developing their self‐management and personal skills to improve their health and wellness, the ECCM proposes developing these skills in a paternalistic way [18]. For example, it is proposed to support the personal and social development of individuals and groups as a way to provide information to enhance life skills. However, populations and individuals do not all have the same capacity to change their lifestyle habits, yet they have experience that can be mobilized to tailor strategies based on their knowledge and willingness to change. The literature shows that involving patient who have been affected by a health problem in therapeutic patient education (TPE) leads to significant differences in their ability to change lifestyle habits and maintain their behaviour over time [19]. This has even been assessed economically as a return on investment of approximately US$80,000 per person [12]. The eCCM [20] highlights the role played by technology in supporting patients with chronic illnesses and considers the importance of engaging patients in different ways (cf. Figure 3). However, the model presents PE primarily in terms of what professionals can bring to patients.

## 3 Methodology

As proposed for the development of the eCCM, the theory derivation process was mobilized to reinforce the patient's voice in self‐management of chronic illness [21]. Theory derivation is a structured set of procedures in which one selects a parent theory or model to guide the development of a new model or theory while drawing on a comprehensive understanding of the current literature. In this article, the CCM was carefully examined, extrapolating components to develop a new model. A draft framework was then developed in light of a review of the literature on the three models identified as most relevant.

A thorough review of the published literature since 2000 was conducted using CINAHL, Medline, OVID, EMBASE, PsychINFO, Science Direct and selected ‘grey’ literature, including government reports. The review involved using the search terms ‘CCM or Chronic Care Model’ and ‘patient engagement’, followed by the specifically identified components of partnership and chronic disease self‐management support (‘coconstruction’, ‘patient expert’, ‘patients’ experience’, ‘experience of care’, ‘peer support’, ‘mediation’, ‘grievance’, ‘complaint’, ‘grief’, ‘claim’, ‘social networking’, ‘patient education’, ‘learning path’, ‘chronic illness’, ‘chronic disease’ and ‘self‐management support’). Selection criteria included review papers, randomized controlled trials, cohort studies, cross‐sectional studies and qualitative studies. The researchers independently identified papers based on their framework, design, sample, measures and fit with PE and chronic illness and added their own research when relevant.

## 4 Results

We identified 354 papers but excluded 64% (129/354) of them due to concerns about study design, sample size and/or methods. Overall, a variety of methods and approaches were used. We organized the literature into three major PE movements that stand out for their potential contribution to the chronic disease management components of the CCM: experience of care through the lens of complaints and mediation in healthcare, patient education and learning paths and patient partnership.

## 5 Experience of Care and Mediation in Healthcare

### 5.1 Definitions

There is a variety of methods for collecting data on the patient experience, including surveys, focus groups, online rankings, shadowing (in which a patient is followed and their journey analysed [22, 23, 24, 25, 26, 27]) and compliments. To evaluate patient experience a source of improvement, the collection of qualitative data, such as complaints, is often underutilized [28]. Grievances collected through a mediation centre offer unique insight into the patient's journey through the healthcare system and enable improvement projects to be developed, inspired by their needs, expectations and the difficulties encountered throughout the course of care [29, 30]. For these grievances to become tools for improvement, creating a mediation space enables professionals and patients to meet and discuss their experiences on a reciprocal basis (Appendix 1) [31, 32, 33]. At the end of the last century, mediation was developed in several European countries, including the United Kingdom, Norway, Belgium, Italy and Switzerland [34]. It quickly became an alternative model of conflict management compared to the treatment of patient–professional conflicts in court, partly because several studies have demonstrated the negative consequences of a legal process on the physician's psyche [8, 35, 36, 37]. In addition, mediation sometimes provides a response to the difficulties encountered by patients and their families [38]. One important motive for patients lodging grievances is to prevent the same thing from happening to others [39, 40]. Counterintuitively, patients who come into conflict with a hospital only seek financial compensation in 7% of cases, whereas they expect explanations 61% of the time [41].

### 5.2 Key Results

Based on grievance analysis, rapid loops for improving care can be conceptualized [36, 37, 42]. They are directly inspired by the institutional shortcomings described and experienced by patients and matter to them. The work involves deconstructing the patient experience and targets the diverse needs that patients encounter throughout their journey [43]. These needs may be relational (e.g., respect and dignity, staff attitude and communication breakdown), or refer to institutional issues (e.g., staffing and resources) or timing and access [31, 39, 40]. Lastly, the grievances may concern clinical aspects of care (e.g., errors in diagnosis or medication) [44, 45]. The aim is to use grievances to develop an improvement project that will prevent the error from recurring and other patients from facing something similar [46, 47, 48]. The patient's experience is then seen as a source of valuable information for improving the integration and continuity of care and services and making the care trajectory as harmonious as possible from the patient's point of view [4].

## 6 TPE and Learning Paths

### 6.1 Definitions

People unfamiliar with TPE may think that it is based on the transmission of adapted health messages from the physician (or health professional) to the patient to strengthen the effectiveness of care or treatment (Appendix 2).

Given the growing importance of TPE, the WHO proposed a definition in 1996: ‘TPE aims to help patients acquire or maintain the skills they need to best manage their life with a chronic illness. It is an integral and permanent part of patient care. It includes organized activities, including psychosocial support, designed to make patients aware of and informed about their illness, care, hospital organization and procedures and behaviours related to health and illness. This aims to help them, as well as their families, to understand their illness and their treatment, to collaborate together and to assume their responsibilities in their own care, with the aim of helping them maintain and improve their quality of life’ [2]. Since then, the patient–physician relationship appears to have become the cornerstone of the ‘educational posture’ [49], which requires a shift by health professionals from a paternalist to a partnership attitude, focused on patients' experience and knowledge. Therefore, the concept of TPE has gradually evolved towards individualized learning pathways that enable people with health problems to consider all facets of their existence and mobilize the expertise of professionals and peers as well as their own. The WHO recently refined its definition by introducing the notion of ‘person’ in the learning process: ‘Therapeutic patient education is a structured person‐centred learning process that supports individuals living with chronic conditions to “self‐manage” their own health by drawing on their own resources, supported by their caregivers and families [50]. It is carried out by trained health professionals and comprises several types of self‐management support interventions. It is adapted to the patient and their condition and continues over the patient's lifetime. It is an integral part of treatment for chronic conditions and can lead to better health outcomes and improved quality of life while making the best use of healthcare services and other resources' [2].

### 6.2 Key Results

The literature in this field is flourishing. Here we will focus on some significant results on improved disease management and psychosocial challenges [51, 52, 53, 54]. A systematic review by Lagger, Pataky, and Golay [51] concludes that TPE interventions are effective in improving biological outcomes, treatment adherence, self‐efficacy and psychological health in patients facing various health problems. A total of 598 studies were analysed, representing approximately 61,000 patients in eight chronic pathologies, with TPE appearing beneficial in 64% of cases, with no demonstrated effect in 30% and negative consequences in 6%. These findings are confirmed in a recent review of the literature focused on TPE interventions that are effective in improving a range of biomedical and psychological outcomes for a variety of chronic disorders [55, 56, 57, 58]. Self‐management support enables empowerment and lifestyle changes that improve clinical outcomes and quality of life and reduce the use of the healthcare system and, in turn, costs [59, 60, 61, 62, 63, 64, 65, 66, 67, 68, 69]. TPE also transforms the professional identity of caregivers from one of teaching and expertise to co‐construction and learning, to better identify and respond to complex situations [53, 70, 71, 72].

## 7 Patient Partnership

### 7.1 Definitions

The patient partnership model is the result of the work carried out by Carman et al. [3] to illustrate different levels of patient involvement. Patient partnership enhanced Carman et al.'s model by integrating other dimensions that were not present in the work carried out in research and teaching (Appendix 3). This also made it possible to specify four specific areas of patients' contributions to justify their involvement: (1) recognition of the experiential knowledge of people with chronic illnesses about living with the disease and using the healthcare system; (2) recognition of patients' role as caregivers to themselves, who influence their own health through their behaviours and the way they take their treatments; (3) full membership on the clinical team for the information needed to understand their life situation and (4) people's ability to make informed decisions about their life project, with the support of professionals (where ‘life project’ refers to the actions, activities and situations that the person wishes to experience or not). This mobilizes values, cultural representations and ethics to help the person define them and put them into practice. The strength of this model lies in its recognition that because patients can be partners in their own care, they can also mobilize their knowledge for others. These patient partners (PP) can intervene at different levels of the healthcare system, particularly by participating equally in co‐constructing decisions [18].

### 7.2 Key Results

In recent years, various research projects have been carried out to evaluate the contribution of this model [73] to the health and social services system [54, 74, 75, 76]. The development of this approach varies according to territorial contexts [62]. In addition, drawing up intervention plans with the person with a health condition, caregivers (if necessary) and the support of an accompanying patient helps avoid misunderstandings with the clinical team, thereby improving the experience for patients [77]. PPs can be involved not only in mental health but also oncology [78], rehabilitation [79] and chronic diseases and conditions [80]. They help improve the quality and safety of care and restore meaning to the work of professionals [81]. They also help reduce patient anxiety and improve quality of life [77]. At the organizational level, the presence of PPs on improvement committees often enables them to find solutions that are pragmatic, cost‐effective and meet their needs. On management committees, they encourage decisions that take patients' experiences into account [82, 83].

## 8 The Expanded Chronic Care Patient–Professional Partnership Model (E2C3PM)

In light of the literature review presented earlier, we propose a new model that better integrates the patient's point of view into chronic disease management while capitalizing on previous models. The aim is to rebalance the balance of power between healthcare professionals and patients (and their caregivers). Indeed, it seems increasingly difficult to find solutions to limit the burden of chronic diseases on the healthcare system without mobilizing patients' knowledge. This model also highlights the crucial role played by patients in the development of integrated care. Because patients are the only ones with an overview of the care pathway, they can also highlight what is preventing a harmonious trajectory, and thus work to ensure that care integration is seamless throughout their journey.

**Figure 4:**
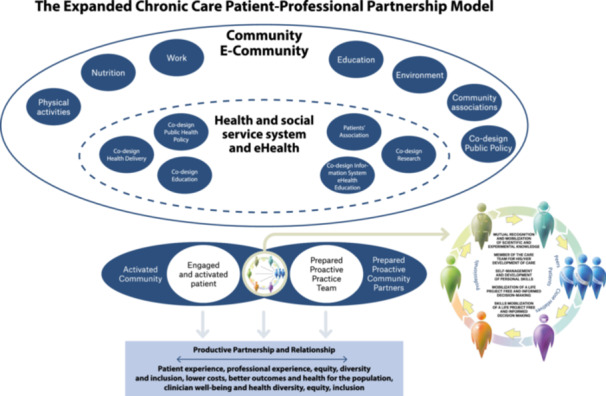
The Expanded Chronic Care Patient–Professional Partnership Model (E2C3PM).

In this new model, the integration of patient empowerment into the E2C3PM underlines the importance of co‐construction with patients at the clinical, organizational and systemic levels in a supportive environment (Figure 4). Here, the concept of coproduction is to be understood as the fact that patients and healthcare professionals have equal weight in the search for solutions. Taking the main categories of the initial CCM model by Wagner, we start with the relationship between patients, peers and healthcare and social providers, then we look at the characteristics of the healthcare and social services system focusing on how to create a supportive community and, finally, at what outcomes should be measured. We have introduced the concepts discussed earlier into these co‐construction dimensions. Table 1 presents a summary comparing the ECCM, the eCCM and the E2C3PM.

**Table 1:** Comparison of the ECCM, the eCCM and the E2C3PM

Dimension	ECCM	Dimension	eCCM	Dimension	E2C3PM
Community	Build public health policy Create supportive environments Strengthen community action	Community—Health systems—eCommunity—eHealth	Self‐management support Delivery system design Clinical decision support Clinical information systems eHealth education	Community and e‐Community	Work Nutrition Physical activities Codesign of public policy Community association Environment Education
Health system	Self‐management: develop personal skills Delivery system design/reorient health services Decision support Information systems			Health system and eHealth	Codesign public health policy Codesign health delivery Codesign education Codesign information system and eHealth education Patients' association Codesign research
Activated community	Informed activated patient	Activated community	Informed activated patient	Activated community	Engaged and activated patient
Prepared proactive community partners	Prepared proactive practice team	Prepared proactive community partners	Prepared proactive practice team	Prepared proactive community partners	Prepared proactive practice team
Outputs	Productive interactions and relationships	Prepared proactive community partners	Complete feedback loop Productive interactions	Prepared proactive community partners	Productive interactions and relationships
Outcomes	Population health outcomes/functional and clinical outcomes	Outcomes	Improved outcomes	Outcomes	Patient experience, professional experience, equity, diversity and inclusion, lower costs, better outcomes and health for the population, clinician well‐being and health diversity, equity, inclusion
Added			Data/Information/Knowledge/Wisdom		Patients as team members

### 8.1 Relationship Between Patients, Peers and Healthcare and Social Providers

#### 8.1.1 Mutual Recognition and Mobilization of Scientific and Experiential Knowledge

For years, the healthcare system has deprived itself of an important asset: patients (and caregivers), particularly those with chronic illnesses. This was on the assumption that there is an asymmetry of knowledge, but above all an asymmetry in the quality of knowledge. Recently, six types of knowledge have been identified as being mobilized by patients with chronic diseases [84, 85]. The first, called *embodied knowledge*, can be likened to knowledge that is felt physically, emotionally and/or cognitively by the body and mind. *Medical knowledge* covers the causes and physiological mechanisms of disease, symptoms, treatment options and types, side effects and benefits and drug interactions. *Self‐management knowledge* concerns the physical, emotional, cognitive and/or behavioural signs associated with the condition and the solutions found on a daily basis to improve them. Knowledge of these signs can be acquired through medical devices, connected objects or self‐awareness. *Relational knowledge* is developed through interaction with the caregivers to whom patients turn within their community to access care, help them manage their condition and support the achievement of their life goals. Patients identify people and organizations both inside and outside the healthcare system that are part of their broader care team. *Navigational knowledge* concerns the workings and logic of the healthcare system, mobilized in procedural and relational ways to access quality care and services at the right time. *Cultural knowledge* refers to the standards, values, symbols, constructions of reality and worldviews that influence the experience of life and illness. This knowledge is not only of cultural codes but also the way they influence preferences, needs and care relationships. All this knowledge can be mobilized by the team as a complement to its scientific, practical and experiential knowledge. Thus, in a patient/healthcare professional relationship based on partnership, reciprocal recognition of knowledge enables optimal apprehension of the complexity of chronic disease management. For some and in specific contexts, this partnership can rely on the ETP to develop or consolidate itself [86].

#### 8.1.2 Member of the Team for a Partnership Relationship

When the knowledge of patients (or their caregivers) is recognized, they become de facto full members of the team. In this relationship of equivalence, the position of healthcare professionals changes. Professionals find themselves accompanying, equipping and enlightening patients as they deal with chronic illnesses. Decisions are made by patients on the basis of their life projects, which can lead to trade‐offs in paradoxical or antinomic situations where treatments or regimens are incompatible. To help patients develop their skills and competencies, peers complement the expertise of other members of the clinical team. They embody a role model into which the patients can project themselves, thereby understanding how their behaviours and treatments can have an impact on their health [81, 87].

Supported in this way to become a partner in their care, patients establish reciprocal relationships with all team members that include reciprocal communication, development of autonomy, informed decision‐making, reciprocal sharing of information relevant to decision‐making, the context of one's life and that of the professional's practice, reciprocal recognition of expertise and the ability to show reciprocal empathy, all supported by an ethical approach [84, 88, 89, 90].

#### 8.1.3 Self‐Management and Development of Personal Skills

To promote health, the target population has to be involved, based on their needs, abilities and knowledge. A health policy to combat smoking can only be designed with input from people who have detailed knowledge of their experience of smoking, including those who have been able to quit and those who have not, and people from different sociocultural backgrounds. Co‐construction with these individuals ensures that the perspective of the people for whom the policy is intended is taken into account from the outset. In the same way, the implementation of a therapeutic education programme on smoking cessation will be co‐constructed with people who have expertise in this field, featuring workshops co‐facilitated by both a professional and a patient expert. Evaluations of TPE programmes carried out in co‐construction with the people affected have shown that their benefits exceeded those achieved by professionals alone [91].

Recently, the Centre of Excellence on Partnerships with Patients and the Public (CEPPP) has developed a promising tool for this purpose: the patient learning pathway (PLP) [50]. PLPs are codeveloped with expert PP, medical and patient organizations, as well as other key stakeholders such as decision‐makers and research groups. They serve as a framework and a reference for improving healthcare and services based on patient competencies, which are addressed as needed, throughout their life journey with illness.

#### 8.1.4 Mobilization of a Life Project

This dimension is new and central to this model. In fact, no decision, even in an emergency or resuscitation situation, should be taken without considering the person's life project. It is no longer professionals who make decisions according to their own frame of reference, but rather people concerned with their own lives. In end‐of‐life situations, advance medical directives are a way to respect the wishes of someone who is no longer able to participate in a free and informed decision. Otherwise, people affected by chronic illnesses express how they wish to direct their life project. For example, a patient suffering from two illnesses, such as ulcerative colitis and diabetes, may have contradictory diet requirements. It is up to the person concerned, and not the doctor, to determine what is more important to control: the diabetes or the digestive problems—based on their life plan.

#### 8.1.5 Free and Informed Decision‐Making

The literature reports the importance of implementing a shared decision [92]. However, our new partnership model proposes going one step further by allowing the person to make decisions informed by other team members, including peers. Decision‐making support tools should be co‐constructed with the main stakeholders, not by professionals alone. It is important to take into account someone's point of view affected by one or more chronic illnesses, as this can lead to the development of more complex tools for integrating several health conditions. By taking patients' real‐life situations as a starting point, and empowering them to make decisions for themselves, patients can become involved in improving their health.

In terms of the community, a general practitioner can work not only in partnership with a health promotion professional but also with citizens who are committed to promoting health and familiar with the best practices for reaching the population.

### 8.2 Healthcare and Social Services System

The model proposes merging the healthcare system with the social services system and adding the eHealth system and social services. This includes elderly care, mental health problems of social origin, addictions and physical and intellectual rehabilitation. It is based on involving patients in all phases (planning, development and implementation) of health intervention/programmes using an implementation science approach, as patient involvement can have a greater impact and exert stronger influence not only on institutions/organizations uptaking and implementing new care models but also its effectiveness/patient outcomes.

#### 8.2.1 Codesign Education for Professionals and Managers

Such a paradigm shift is impossible without a complete overhaul of the training of professionals and managers dealing with chronic illnesses. Initial and ongoing training courses are needed to enable these professionals and managers to work in partnership. The aim is to train them to work on interdisciplinary teams and ensure that faculties of medicine and other health and social services disciplines include PP in all their training, with patients coaching professionals, participating in curriculum development and helping select future professionals. The model used at Université de Montréal could be a source of inspiration [4]. In this model, patients are involved in the training given to health and social services managers so that they take better account of patients' experience in their decision‐making processes.

#### 8.2.2 Codesign Information System and eHealth Education

The proposed model fully applies what was presented in the eCCM [20]. We emphasize the importance of having a clinical information system managed by the patients themselves, with healthcare professionals providing additional information, as in the Haemophilia model [90]. The patient health record (PHR) can help patients' engagement by facilitating preparation for appointments and tracking laboratory results and diagnostic studies. They can be involved in preventive care and screening and suggest treatments to their providers [93, 94]. The use of a PHR can promote an informed, activated patient in self‐management support and productive interactions [95, 96, 97, 98, 99]. In terms of both medical records and databases, such developments present new challenges, including the quality of data collection, accessibility to technology and data security [100].

In addition, for chronic disease management, more and more e‐health tools are available for patients (eMessage, eCommunity, health education, etc.). Therefore, developing eHealth literacy skills in accessing online information is becoming a necessity [101]. The PLP approach developed by the CEPPP mentioned earlier proposes a method to respond to these new challenges inherent to e‐health tools [50].

#### 8.2.3 Codesigning Public Health

The population‐based approach, which was first developed in the 1990s, enables the various players who can act on the social determinants of health to work together to improve the health of a population [12]. By mobilizing, across a service territory, the available resources that act on the social determinants of health, and by coordinating the supply of services to a given population, enables the population to participate in the creation of an environment conducive to its health, with the support of the relevant institutions. These initiatives can take a variety of forms, such as citizen spaces dedicated to a given population, neighbourhood round tables, networks of citizen scouts or a healthcare community [102, 103, 104]. Neighbourhood round tables bring together all the local players who can play a part in improving the quality and conditions of life in a neighbourhood. This includes health and educational institutions, the police, city departments, shopkeepers, workers, professionals, representatives of associations and citizens. They help find solutions to complex issues such as chronic disease prevention. Lastly, networks of citizen scouts are springing up all over the world. These are networks of citizen volunteers, ‘relay workers’ with psychosocial experience, who act in their communities to support and care for them. They are usually trained in mental health prevention and promotion and in resolving situations of distress. Much like the Healthy Communities/Healthy Cities movement worldwide [105], the caring community movement aims to bridge the gap between health and community by integrating peers into frontline teams [106, 107, 108].

#### 8.2.4 Codesign of Health Policy

Public health policies are still too often conceived and drafted by experts working at the political (cabinet), administrative (government departments), academic (research centres and universities) or practice (facilities) levels. The model proposes that discussions of these policies should systematically include PP and patient association representatives to ensure that their needs and ideas for meeting those needs are taken into account.

#### 8.2.5 Codesign Research

To support the model and ensure that it meets patients' needs, research strategies must be co‐developed with patients. Frameworks for evaluating patient involvement in research help research teams assess the PE process. They also help ensure proper preparation for partnership and its contribution throughout the research process. Such a framework also enables research partnerships to be continuously assessed and improved and guides the planning of partnered research projects, including knowledge transfer [109, 110, 111, 112, 113]. Another avenue is the deployment of research chairs cochaired by career researchers and patients. Several such initiatives have been launched in recent years to address chronic diseases [114, 115].

### 8.3 Create a Supportive Community

The proposed model fully incorporates the ECCM, including the importance of social support and a health and social services system.

### 8.4 Outcomes

To measure the value of healthcare systems, the Institute for Healthcare Assessment proposes assessing health system outcomes through the Quintuple Aim. The Quintuple Aim refers to effects on patient experience; professional well‐being and health; diversity, equity and inclusion (DEI); lower costs; and better outcomes for the population [116].

More and more countries are routinely monitoring patient‐reported outcomes or experience measures (PROMs and PREMs) [117, 118, 119, 120]. International organizations such as Eupati [121], Ichom [122] and the Centre for Advancing Health Outcomes are part of this movement [123].

Regarding professional well‐being and health, various dimensions need to be considered (participative and shared leadership, managers concerned with the needs of professionals, respect for balance between personal and professional life and training for professional and personal development). A model has been proposed to better grasp these various dimensions [124, 125]. However, although some tools stand out in the literature, they are not yet being used for international comparisons [126].

Measuring DEI is now a must in the health and social services system [127]. This is particularly true for chronic diseases whose development appears strongly linked to the socioeconomic conditions [128]. This is also apparent during student training, where curricula are developed to raise awareness of how to put these concepts into practice [129].

In addition, the economic dimension is central to evaluating the model's effects due to limited resources. Part of the value‐based healthcare movement [130, 131], this approach requires financial quality data as well as data on population and clinical outcomes, patient‐reported outcomes and patient experience. It is important to take consistent measurements using validated instruments. Various economic studies can be performed: assessments of direct remuneration and costing every input consumed in treating a particular patient [132, 133]; gross costing, which assesses the total budget for specific services, such as hospital stays, by grouping encounters with similar usage profiles [134]; and assessing value through the use of quality‐adjusted life‐year [135].

Lastly, the model seeks to help patients with chronic diseases achieve better health outcomes [136] (e.g., in smoking‐related mortality, obesity rates, mental health, life expectancy in good health and health disparities) and a state of well‐being, meaning that they feel good, experience positive emotions and can give their best in the various spheres of their lives [137].

There are different ways to measure population outcomes. The first is mortality outcomes, through indicators related to death and survival rates. They reveal premature deaths and disparities between populations. Morbidity outcomes focus on the incidence and prevalence of diseases, conditions and disabilities, emphasizing the disease burden and a population's overall health status. Health‐related quality of life (HRQoL) and patient‐reported outcomes capture the impact of health on individuals' overall well‐being, including physical, mental and social aspects. These outcomes help identify the reality experienced by the patient. The indicators are increasingly used to fine‐tune public health policies, which are co‐designed by decision makers, civil society players, patients and citizens.

## 9 Conclusion

Over the past few years, the literature has proposed various models for the integrated management of chronic diseases. This began in 1998 with the CCM proposed by Wagner [16], which highlighted the fact that chronic disease management requires a relationship between patients and professionals in which everyone must be involved. The model also highlighted the importance of context in this relationship. This model has evolved into the ECCM, which takes into account the intrinsic role played by the social determinants of health in influencing individual, community and population health and the eCCM, to better take into account the contribution of technology to chronic disease management. However, in the age of the Patient Revolution [138], these models continue to see professionals as the knowers and fail to take into account the essential role played by patients and citizens in promoting their health, as well as the importance of their involvement at all levels. To better understand this essential contribution, a new model is proposed, based on the recognition of people's experiential knowledge and the co‐construction methodology.

Applying this model should help take better account of the complexity of chronic illnesses to improve integrated care, which is defined as ‘a coherent set of methods and models on the funding, administrative, organizational, service delivery and clinical levels designed to create connectivity, alignment and collaboration within and between the cure and care sectors. The goal of these methods and models is to enhance the quality of care and quality of life, consumer satisfaction and system efficiency for people by cutting across multiple services, providers and settings’ [14]. Integrated care includes not only care and services but also the various determinants of health and how to reach a mutually beneficial settlement among all the stakeholders involved.

## 10 Future Research and Directions

The model we propose to promote the integration of care for the prevention and management of chronic diseases should help stimulate debate around patient and citizen participation at all levels of the healthcare system and in the community. Evaluations will be needed, in particular as part of participatory research, to continue developing best practices co‐constructed by the various players.

## Author Contributions

**Marie‐Pascale Pomey:** conceptualization; methodology; formal analysis; writing–original draft; writing–review and editing; supervision. **Béatrice Schaad:** conceptualization; formal analysis; writing–original draft; writing–review and editing. **Aline Lasserre‐Moutet:** conceptualization; formal analysis; writing–original draft; writing–review and editing. **Philip Böhme:** formal analysis; writing–original draft; writing–review and editing. **Mathieu Jackson:** formal analysis; writing–review and editing.

## Ethics Statement

This is a review article, so it does not require ethics committee approval.

## Conflicts of Interest

The authors declare no conflicts of interest.

## Supporting information

Supporting information.

Supporting information.

Supporting information.

## Data Availability

The data that support the findings of this study are available from the corresponding author upon reasonable request.
