# Supplementary material for: Towards a New Integrated Model for Taking Into Account the Experiential Knowledge of People With Chronic Diseases, Integrating Mediation, Therapeutic Education and Partnership: The Expanded Chronic Care Patient–Professional Partnership Model
Source: Health Expect. 2024 Oct 7;27(5):e70054. doi: 10.1111/hex.70054 (PMC11456963; doi:10.1111/hex.70054)
Supplement: Supplementary file 1 — Supporting information. [file HEX-27-e70054-s001.docx]

**Appendix 1. Example of integrating the patient experience via a Mediation Centre**

| In April 2012, the Centre Hospitalier Universitaire Vaudois (CHUV), a teaching hospital located in Lausanne, Switzerland, opened a Mediation Centre for Patients, Relatives and Professionals (MCP) that has four missions:  *1) To be a good listener with hospital users who have encountered problems in the course of their care.* People wishing to share one or more experiences can come to the MCP without an appointment or contact the mediators by email or telephone. The mediation team notes the salient features of their care experience and enters the data into a software program, illustrating the comments with verbatim transcripts. These testimonials are strictly confidential.  *2) To work at rebuilding the bond of trust between healthcare professionals and patients/relatives.* There are three ways the MCP works to restore the bond of trust: (1) if the patients or their close relatives simply wish to talk about the problems they had, it is up to the mediators to relay this testimony to the department managers so that they will be aware of it and, if necessary, make adjustments to their practices; (2) the patients or their close relatives can contact the mediation team for help in formulating their grievances and return to the care teams on their own to settle their differences with them; and (3) the patients or their close relatives may meet again with the teams with which they have had problems as part of a mediation session organized and conducted by a conflict specialist.  *3) To systematically collect data on user experience in a software program.* Each situation is classified into one of three main categories: interpersonal (e.g., communication, attitude of caregivers), clinical (e.g., difficulties related to quality and safety of care) and managerial (e.g., administrative difficulties or links with external service providers).^8^ This taxonomy of hospital grievances is based on a meta-analysis of 59 international studies covering 88,000 hospital grievances, and is internationally recognized. ^7^  *4) To present the results to various levels of the organization, with a view to implementing improvement projects.* The synthesis of these testimonials is then shared anonymously and aggregated by team, illustrating the statistics (number of grievances by theme) with verbatim transcripts. The presentations do not identify the patient or the professional concerned. Results can be presented by dissatisfaction theme, by professional category, by clinical specialty, or for an entire care site. A third of the patients and their relatives who turn to this mediation space do so in a spirit of health democracy to participate, through their testimonies, in improving hospital processes. After 12 years in operation, the MCP has collected some 6,000 experience accounts, providing a wealth of information on users' experiences and guiding the development of several practice improvement projects each year. |
| --- |
