# Supplementary material for: Towards a New Integrated Model for Taking Into Account the Experiential Knowledge of People With Chronic Diseases, Integrating Mediation, Therapeutic Education and Partnership: The Expanded Chronic Care Patient–Professional Partnership Model
Source: Health Expect. 2024 Oct 7;27(5):e70054. doi: 10.1111/hex.70054 (PMC11456963; doi:10.1111/hex.70054)
Supplement: Supplementary file 2 — Supporting information. [file HEX-27-e70054-s003.docx]

**Appendix 2 The diabetes teaching and treatment unit at Geneva University Hospitals (HUG).**

| In 1975, Professor JP Assal^62,63^ set up the Diabetes Teaching and Treatment Unit at the Geneva University Hospitals (HUG). From the outset, the unit welcomed diabetic patients, then people suffering from metabolic disorders and obesity. Renamed the Therapeutic Patient Education Unit in 2022, the structure helps patients learn to live with their disease and their treatments. It is made up of an interdisciplinary team of doctors, nurses, dieticians, psychologists, educationalists, art therapists and patients. Recognition of patients' lived experience and experiential knowledge is the cornerstone of the support model for people living with one or more long-term illnesses. In 1983, the unit was recognized as a WHO Collaborating Centre for reference and research in the field of therapeutic patient education and long-term follow-up strategies for chronic diseases. In 2023, the Swiss Federal Office of Public Health included therapeutic patient education in its new concept of self-management support.^64^ Representatives of the people concerned and their families have contributed to the content, to ensure that the concept has not been developed *for* but rather *with* the people concerned. The concept of self-management is based on the partnership relationship established between the people concerned and their professionals, on health promotion in the sense of salutogenesis, and on the ability to accompany people on their educational journey.^61^ |
| --- |
