# Supplementary material for: Towards a New Integrated Model for Taking Into Account the Experiential Knowledge of People With Chronic Diseases, Integrating Mediation, Therapeutic Education and Partnership: The Expanded Chronic Care Patient–Professional Partnership Model
Source: Health Expect. 2024 Oct 7;27(5):e70054. doi: 10.1111/hex.70054 (PMC11456963; doi:10.1111/hex.70054)
Supplement: Supplementary file 3 — Supporting information. [file HEX-27-e70054-s002.docx]

**Appendix 3 The Montreal Model**

| The Montreal Model, so named after the Josiah Macy Foundation in the United States in recognition of the originality of the approach, was developed at the Université de Montréal's Faculty of Medicine. To encourage interdisciplinarity among health and social services professionals, the 13 programs that train these professionals have created an intercurricular activity to promote understanding and collaborative work among the various disciplines that rub shoulders in Quebec's health and social services system. This initiative has made it possible to bring the patient into the interdisciplinary team, by recognizing patient expertise as complementary to that of the professionals. Over the past 10 years, various patient partnership (PP) initiatives have been implemented in the Canadian healthcare system, training, research, public health and the community. Here follows just a few examples of the innovations that have been implemented and evaluated.  ***The health and social services system.*** All health and social services establishments in Quebec have departments responsible for PPs. These departments select patient partners, train and coach them so that they can intervene at different levels of the institution (strategic, management and clinical), and respond to requests from professionals and managers wishing to work with PPs.^81^ Recently, healthcare reform legislation was adopted in Quebec. The implementation committee consists of various healthcare professionals, including a PP.^82^  ***Training*.** The Faculty of Medicine at Université de Montréal is recruiting PPs to participate in the training of healthcare professionals. They sit on medical student selection committees, help recruit residents in certain specialties, coach medical and nursing students during their studies, and take part in clinical ethics workshops. The CanMed^83^ assessment of physicians' skills takes into account their ability to establish partnerships with patients. What is more, the Royal College of Physicians and Surgeons of Canada's final examination jury now includes patients. Future managers are increasingly trained by patients, as proposed by the School of Public Health at Université de Montréal.^84^ PPs are also included in the continuing education activities offered to healthcare professionals. ^18^  ***Research*.** Canadian granting agencies (e.g., Canadian Institutes of Health Research; Fonds de recherche du Québec - santé (FRQS)) have developed policies favouring the integration of patients at various levels of research. The CIHR, for example, recommends that research projects be co-constructed with a person affected by a chronic health condition (PACHC) and that they serve as a co-investigator,^85,86^ and it recruits PPs to sit on its application review committees and governance committees.^18^  ***Health policies*.** When the Quebec government decided to draft a partnership framework, it chose to co-construct it with PPs and various stakeholders in the health and social services network.^87^ Also, during the COVID-19 crisis, a national ethics committee was formed with PP members. This committee worked on a protocol for prioritizing care in intensive care units.  ***Public health***. Civic involvement takes many forms, including the Aire Ouverte program for vulnerable young people aged 12 to 25 years.^88,89^ This scheme provides health and social services to young people who do not make use of traditional health networks. Every month, a committee made up of young volunteers and staff members from the structure defines the program's areas of focus and the priority actions to be carried out, and makes an assessment of past actions. In this way, the population targeted by the program is widely consulted, and thus becomes a major actor in change in public health prevention efforts. ^4^ |
| --- |
